# Supplementary figures and images for: ChromaFactor: Deconvolution of single-molecule chromatin organization with non-negative matrix factorization
Source: PLoS Comput Biol. 2025 Feb 18;21(2):e1012841. doi: 10.1371/journal.pcbi.1012841 (PMC11849981; doi:10.1371/journal.pcbi.1012841)

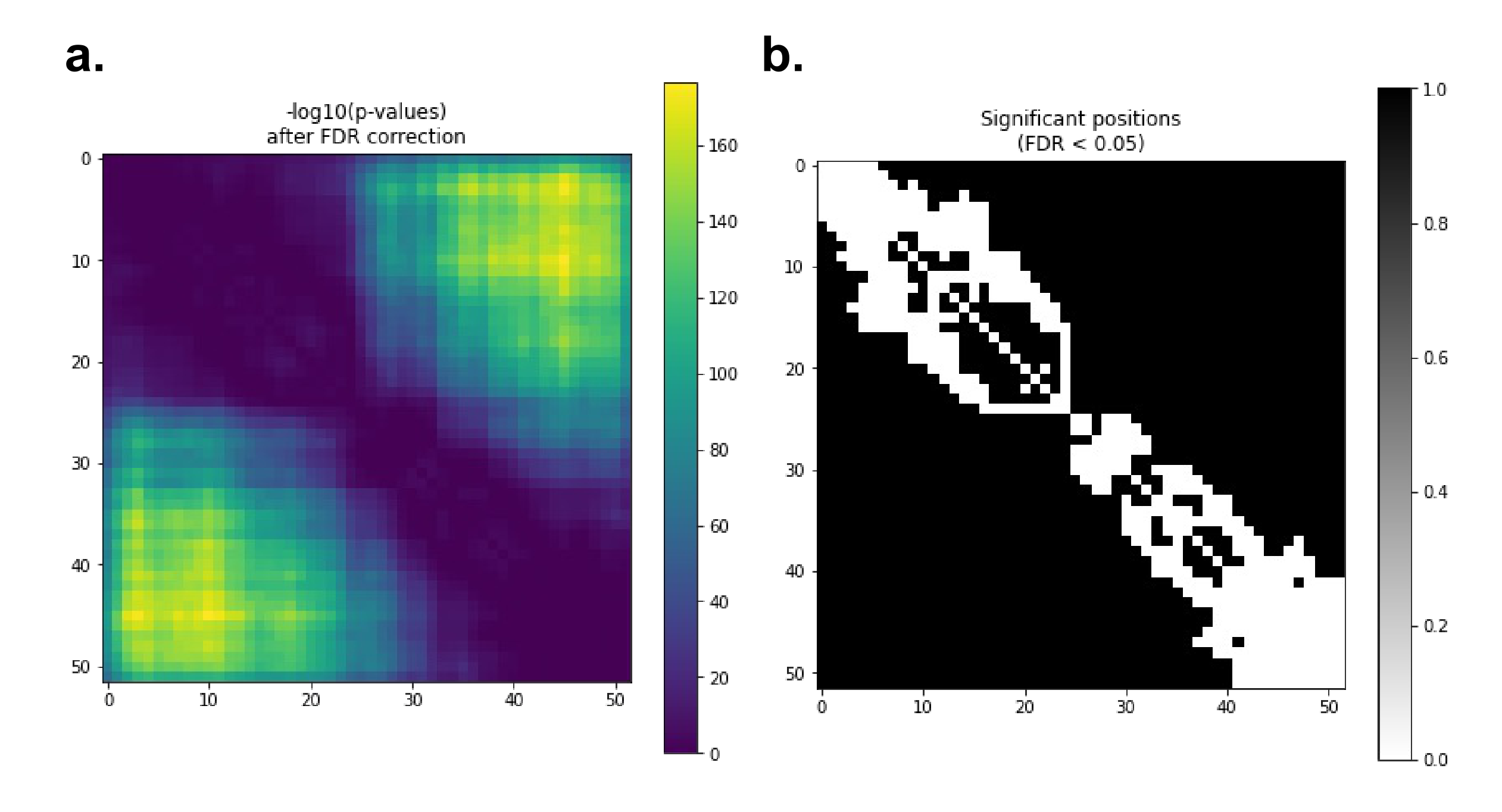

Supplement: S1 Fig — A. Heatmap showing -log10(p-values) from Mann-Whitney U tests comparing contact values at each matrix position between transcribed and non-transcribed cells shown in Fig 1, with Benjamini-Hochberg correction for multiple testing. Darker colors indicate more significant differences. B. Overlay of significant positions (FDR < 0.05, outlined in white) on the median difference map from Fig 1a. (TIFF) [file pcbi.1012841.s001.tiff]

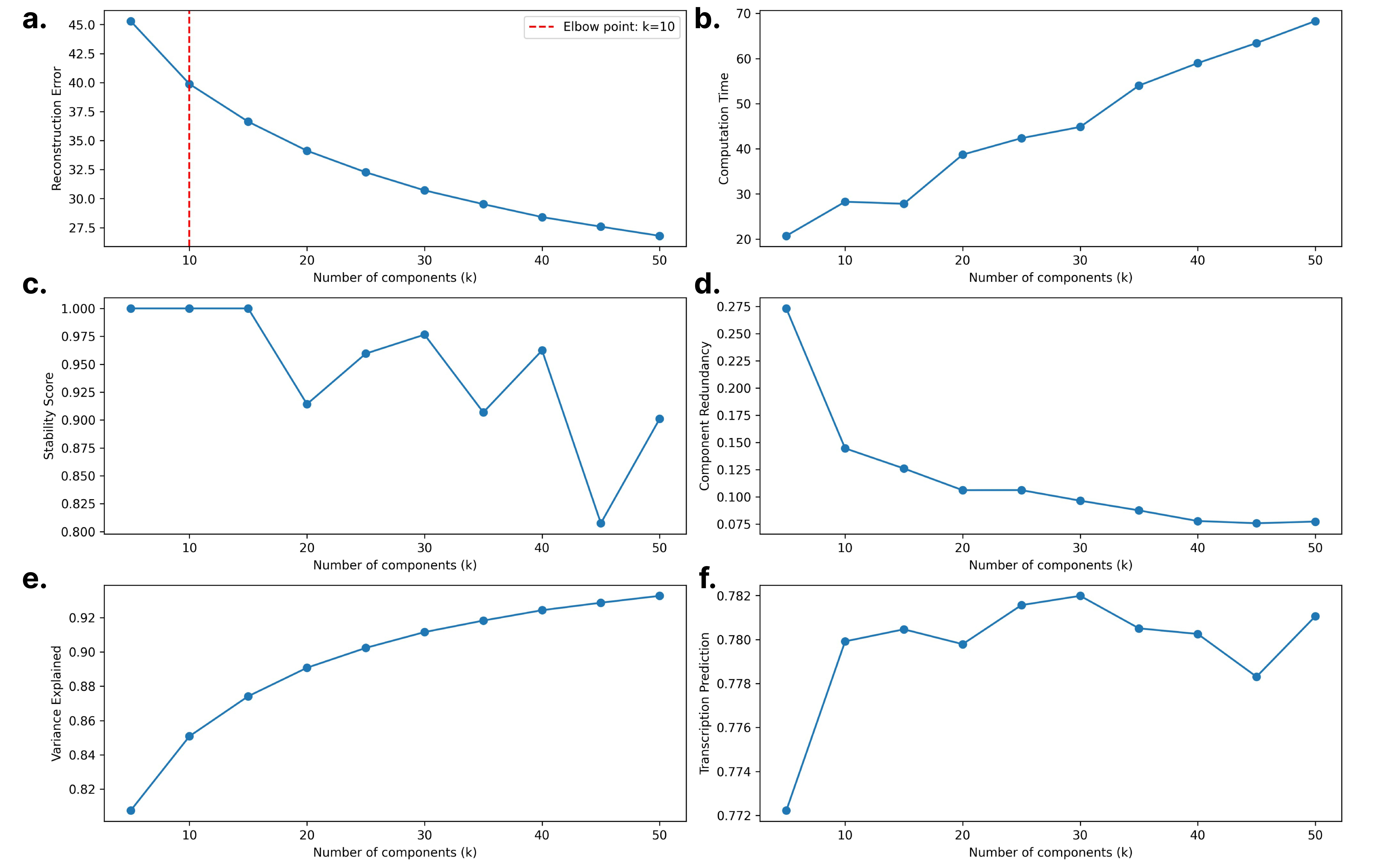

Supplement: S2 Fig — A. Reconstruction error (Frobenius norm) between original and NMF-approximated matrices across different k choices. The red dashed line indicates the elbow point where additional components yield diminishing returns. B. Computational time (in second) required for NMF decomposition. C. Component stability measured as the average correlation between components across multiple random initializations, with higher values indicating more stable templates. D. Component redundancy calculated as the mean absolute correlation between component pairs. Lower values suggest components capture distinct patterns. E. Variance explained showing the fraction of total variance in the data captured by k components. F. Transcription prediction accuracy using random forest classification with component weights as features, demonstrating the biological relevance of the decomposition. (TIFF) [file pcbi.1012841.s002.tiff]

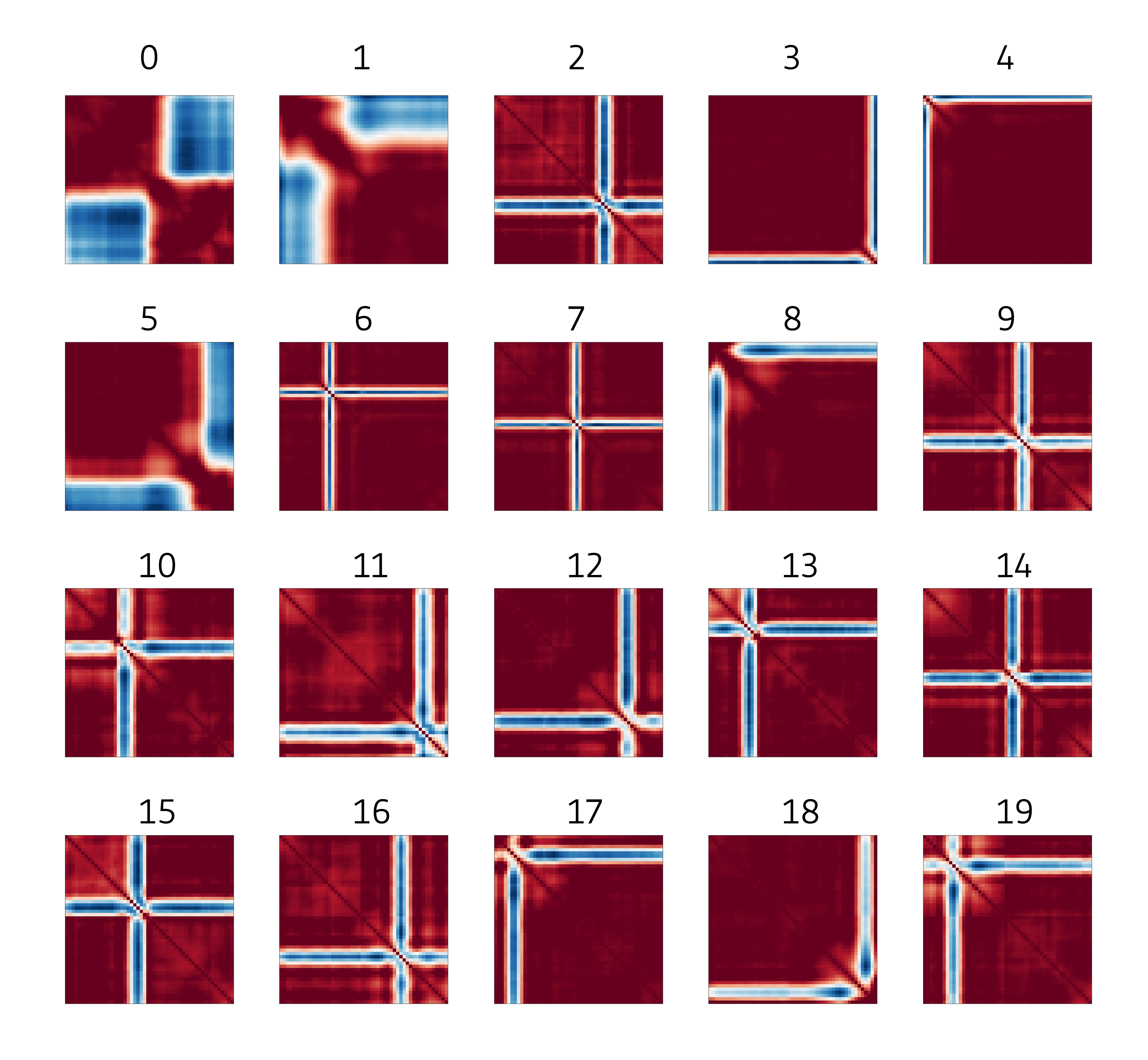

Supplement: S3 Fig — All 20 components generated by applying NMF across the cells at this locus from the Mateo et al. dataset. (TIFF) [file pcbi.1012841.s003.tiff]

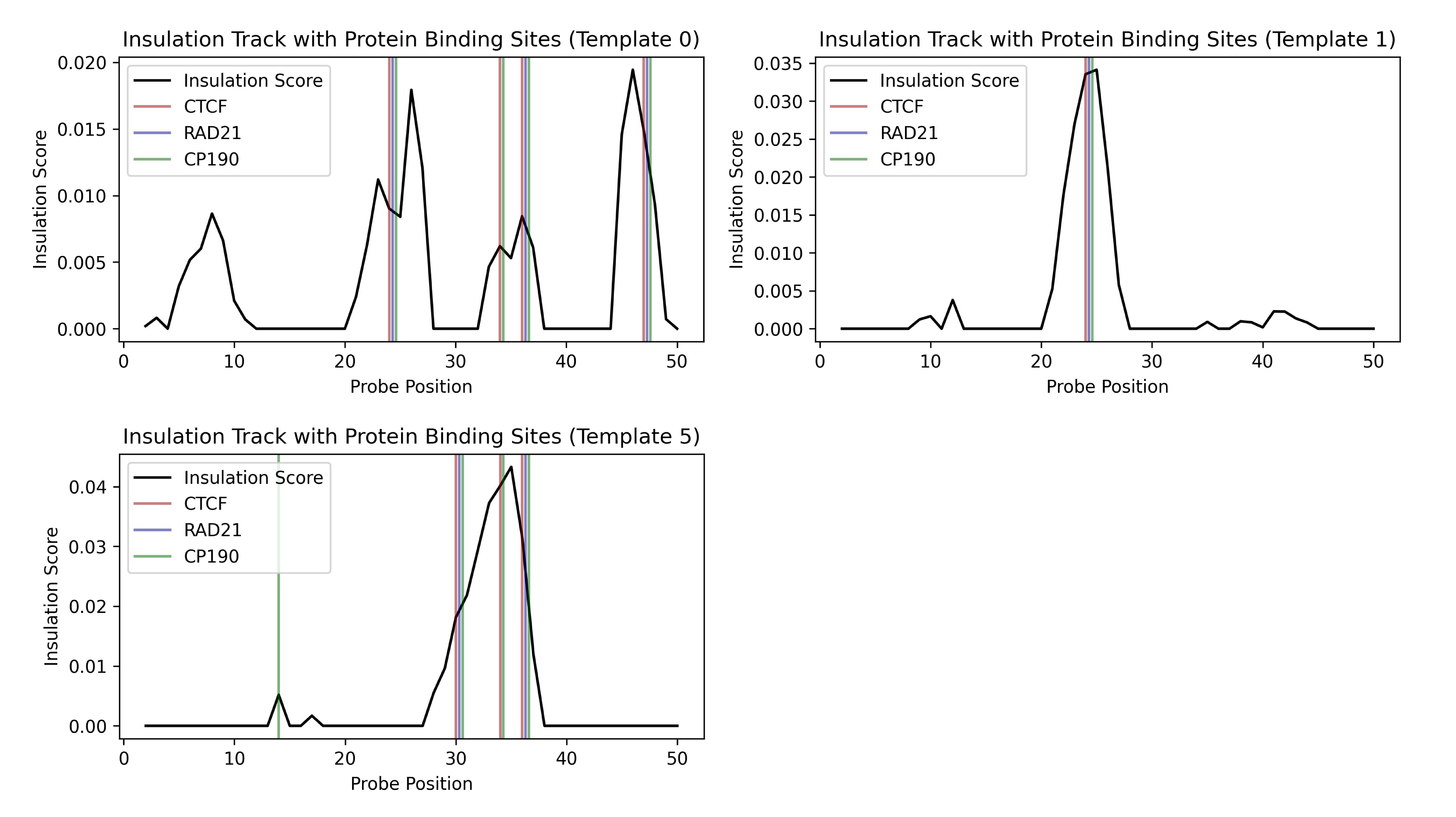

Supplement: S4 Fig — Insulation scores computed across templates 0, 1, and 5 overlaid with ChIP-seq binding locations for CTCF (red), Rad21 (blue), and CP190 (green). Vertical lines indicate protein binding sites at locations where template insulation score exceeds 0.05. Multiple lines at the same position indicate concurrent binding of different proteins. (TIFF) [file pcbi.1012841.s004.tiff]

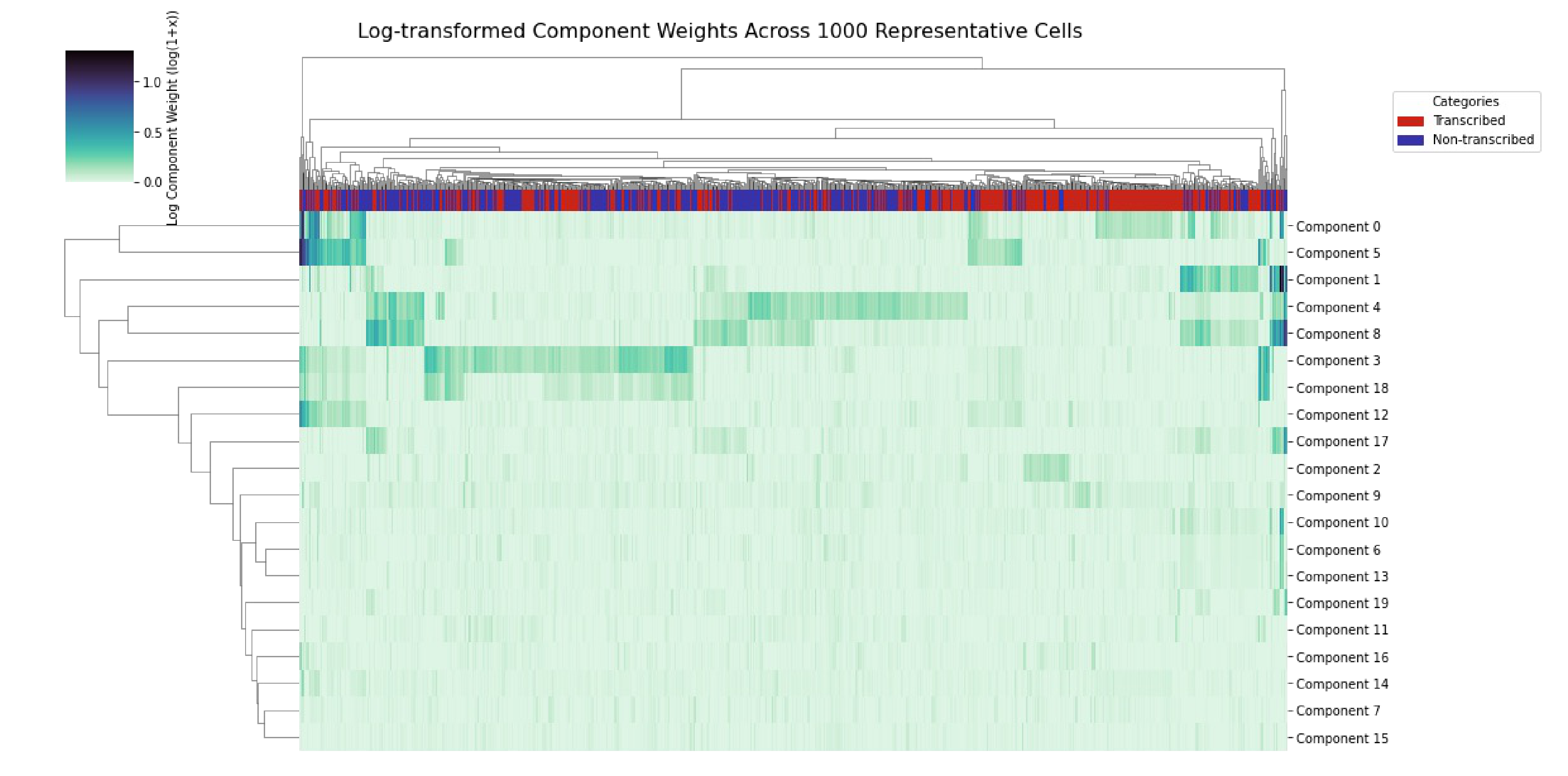

Supplement: S5 Fig — We selected a balanced subset of 500 transcribing and 500 non-transcribing cells with the highest component contributions. Rows (components) and columns (cells) were hierarchically clustered. Cells are labeled by transcriptional state (transcribing/non-transcribing), revealing patterns of component weights that correlate with transcriptional activity. (TIFF) [file pcbi.1012841.s005.tiff]

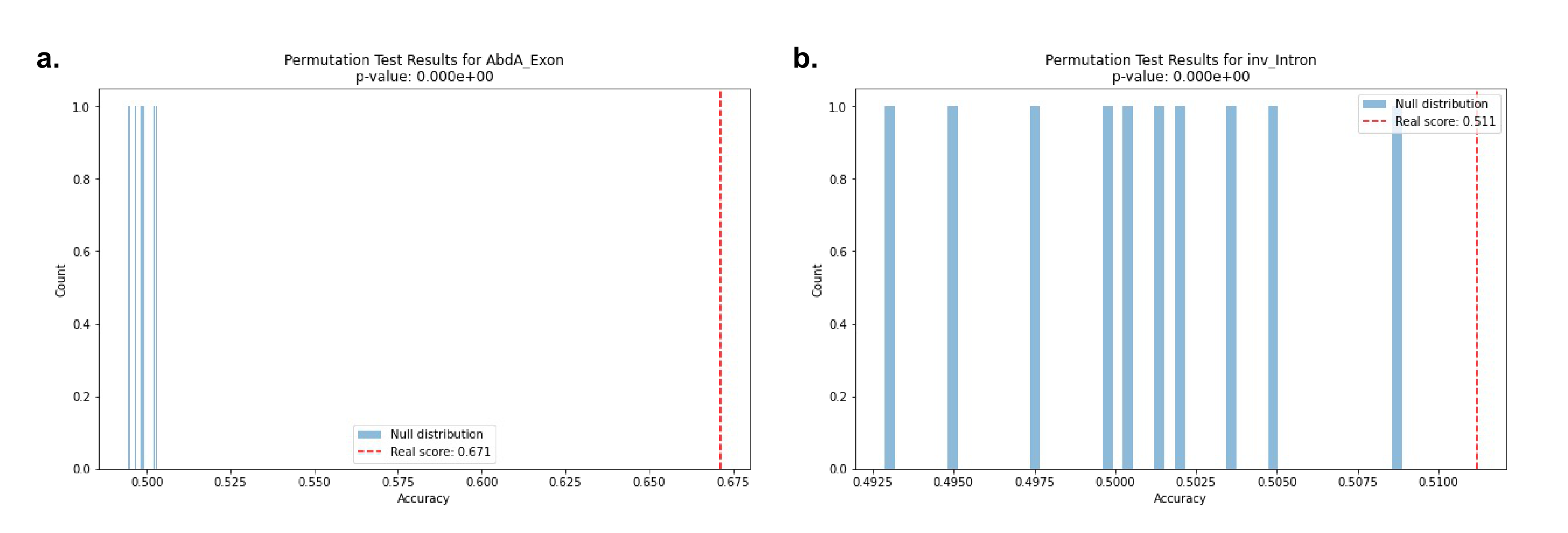

Supplement: S6 Fig — A. Random forest prediction accuracy for Abd-A transcription using original data (0.671) compared to 10 permutations with randomly shuffled transcription labels (mean 0.498 ± 0.003, p < 2.2×10^−16^). The significant drop to random chance performance demonstrates that the original predictive signal is not arising by chance. B. As a negative control, random forest prediction accuracy for inv_Intron transcription using original data (0.511) compared to 10 permutations with randomly shuffled labels (mean 0.500 ± 0.002). Both original and permuted performances are effectively equivalent to random guessing, confirming that templates show specificity for relevant genomic loci. (TIFF) [file pcbi.1012841.s006.tiff]

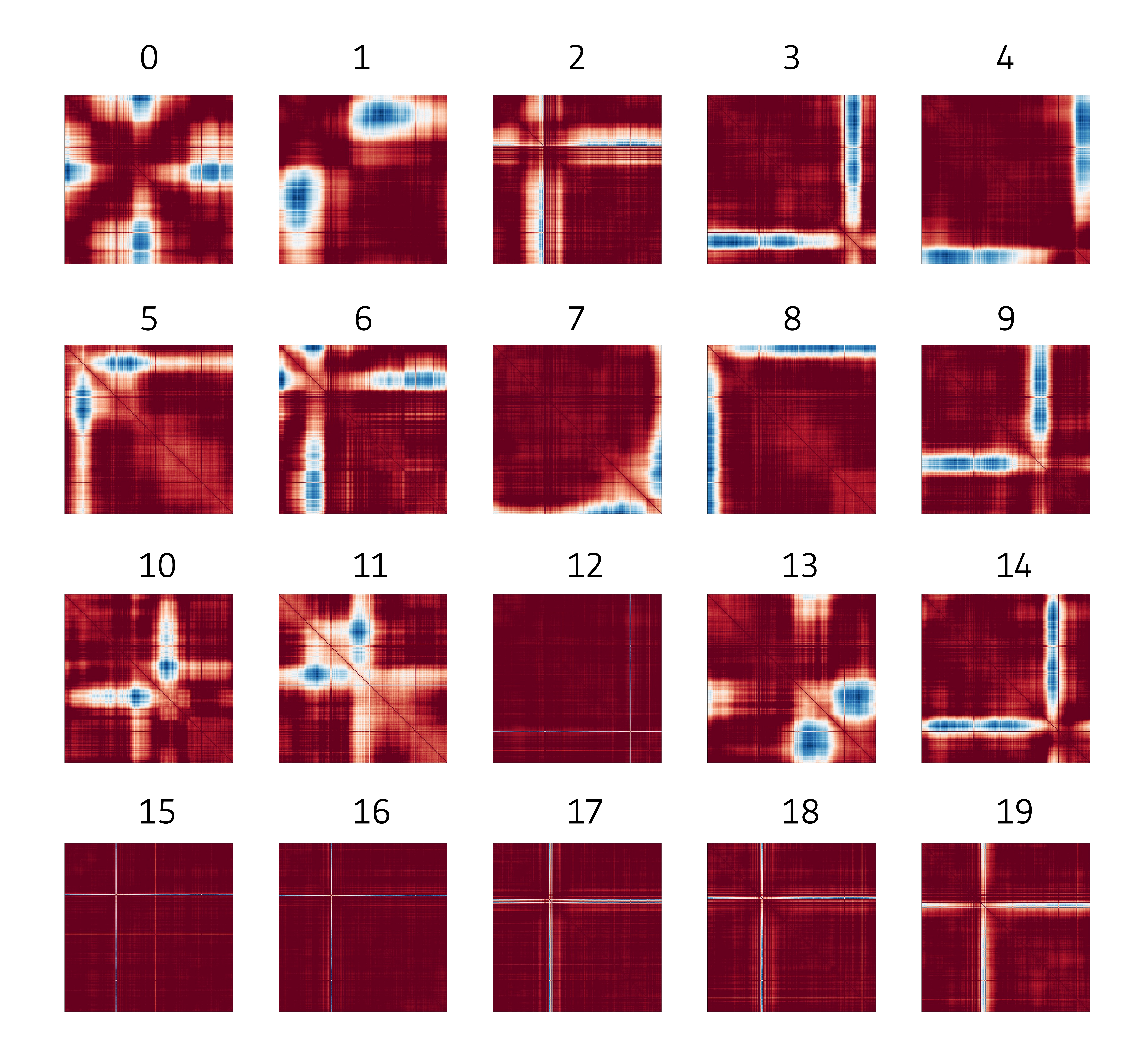

Supplement: S7 Fig — All 20 components generated by applying NMF across the cells at this locus from the Su et al. dataset. (TIFF) [file pcbi.1012841.s007.tiff]
