# Supplementary material for: ChromaFactor: Deconvolution of single-molecule chromatin organization with non-negative matrix factorization
Source: PLoS Comput Biol. 2025 Feb 18;21(2):e1012841. doi: 10.1371/journal.pcbi.1012841 (PMC11849981; doi:10.1371/journal.pcbi.1012841)
Supplement: Attachment — Submitted filename: Response.docx [file pcbi.1012841.s009.docx]

Responses to Reviews

We thank the reviewers for their thoughtful feedback on our manuscript. Below is our point-by-point response to the reviewer comments / questions with the reviewer comments in **black font** and our replies in **blue font**. Changes to the manuscript are denoted in the manuscript file using **red font.**

**Comments to the Authors:
Please note here if the review is uploaded as an attachment.**

**Reviewer #1:**

ChromaFactor describes a non-negative matrix factorization method on single-cell chromatin contact matrices that deconvolves contact matrices to primary components of contact variations and their cellular contribution. Such components are then associated with cellular transcriptional profiles. While the general idea of associating patterns of chromatin contacts with transcription is interesting, several major issues need to be addressed:

We are glad that the general idea behind ChromaFactor is deemed interesting and hope that our revision fully addresses the issues raised.

1. The authors demonstrate that their method provides a clearer picture than bulk-level analysis through visualization, which is not surprising given that the bulk-level analysis ignores existing single-cell variations. A more acceptable baseline would be to derive transcription-associated contact patterns between the transcribed vs non-transcribed cell populations and compare that with the result of ChromaFactor.

Thank you for this thoughtful suggestion about baseline comparisons. We agree that directly comparing transcribed and non-transcribed cell populations is important. Towards this, we included an analysis in the initial submission (Figure 1a) that visualizes the median contact patterns between transcribed and non-transcribed cell populations, along with their difference. This comparison establishes that while clear differences exist at the population level, these patterns are difficult to discern in individual cells. We used this analysis as motivation for ChromaFactor since, as you note, bulk-level analysis ignores single-cell variation and a method is needed to identify which subpopulations of cells drive these bulk differences.

To more quantitatively address the reviewer’s suggestion, we have now added a rigorous statistical analysis comparing contact patterns between transcribed and non-transcribed cell populations. Specifically, for each position in the contact matrix, we performed a Mann-Whitney U test comparing the distribution of contact values between transcribed and non-transcribed cells, with Benjamini-Hochberg correction for multiple testing. This analysis validates the significance of the contact pattern differences shown in Figure 1a (see **Supplementary Figure 1**). We have updated the Results and Methods with this additional analysis.

While this statistical analysis is valuable, we note that we had originally refrained from including it because treating cells as independent examples is a questionable assumption given that they were drawn from the same sample and likely have a complex correlation structure. If the reviewer has a different type of pattern analysis in mind as a baseline comparison, we would be very open to implementing additional comparisons that would strengthen this evaluation.

2. Other matrix decomposition methods exist on single-cell Hi-C datasets, such as Higashi (Zhang et al. 2022) or Topic modeling (Kim et al. 2020). What is the methodological advantage of ChromaFactor compared to other matrix factorization methods? It would be interesting to compare ChromFactor’s reconstruction error and components with those methods.

Thank you for suggesting these comparisons. While Topic modeling, Higashi and ChromaFactor all analyze single-cell chromatin organization data, there are several fundamental differences that make direct comparisons challenging:

1. Input Data Characteristics:

- In our manuscript, we used ChromaFactor to analyze dense imaging-based contact matrices (approximately 60-80% non-zero entries) at very high resolution (3kb) for individual genomic loci (<10Mb in the loci we investigated)
- Topic modeling and Higashi were specifically optimized for extremely sparse single-cell Hi-C data (typically <5% non-zero entries) at much lower resolution (tens of kb or more)
- Applying Higashi to our dense matrices would be computationally inefficient and potentially unstable, as its architecture assumes and exploits sparsity

2. Genomic Scale & Training:

- Our analysis focuses on a 156kb locus with 52 probes on one chromosome
- Higashi requires multiple chromosomes for proper training/validation split to avoid overfitting
- Training Higashi on a single locus would likely lead to overfitting due to insufficient data for its cross-chromosome validation approach

3. Alternative Comparison:

One might consider applying ChromaFactor to single-cell Hi-C datasets. However, this would require first imputing the sparse Hi-C data (likely using Higashi) before applying ChromaFactor to the imputed matrices. This circular dependency would make interpretation of any performance differences problematic.

To summarize, Higash and Topic Modeling are primarily focused on genome-wide imputation, clustering, and cell-type identification, while ChromaFactor was designed for hypothesis generation about regulatory mechanisms at specific loci.

Despite these differences, we attempted to run both Higashi and Fast Higashi on the datasets analyzed in our manuscript. However, we ran into issues adapting these tools to single-locus, high-resolution imaging data. We were unable to get these tools to run with any data that has a low number of bins and a high resolution, and we traced this to the internal pooling operations that are designed to reduce the dimensionality of the input data and quickly reach an expected tensor size below zero for the datasets in this study. We then attempted to upsample the imaging data sets to overcome this issue, but this also failed to run successfully. We were able to run Fast Higashi on a single-cell Hi-C dataset composed of ~4000 cells using a resolution of 1Mb. So, it seems that the Higashi tools require genome-scale data.

Based on these analyses, we conclude that the fundamental differences in data characteristics, scale, and methodological goals make ChromaFactor and Higashi complementary rather than competing approaches. Hence, we respectfully refrain from performing a benchmarking type comparison. Going forward, we are excited about the potential of combining these methods - using Higashi's strengths in handling sparse genome-wide data with ChromaFactor's ability to extract interpretable components at loci of interest.

3. Is the method applied to a single chromosome or across all chromosomes?

Thank you for this question. ChromaFactor is designed to analyze chromatin conformation at individual genomic loci rather than across all chromosomes simultaneously. In our study, we demonstrated its application at two specific loci: the bithorax complex (BX-C) in Drosophila and a 10 Mb region containing the HLCS gene in human IMR90 cells. This locus-specific approach allows us to focus on understanding the relationship between local chromatin organization and transcriptional activity of specific genes of interest. While the method could theoretically be applied to larger regions or multiple chromosomes, we focused on individual loci to maximize interpretability and computational efficiency.

The reviewer’s comment helped us to realize that the locus level focus on ChromaFactor was sufficiently emphasized in our manuscript. To address this, we clarified the size of the analyzed regions in the main text, as well as in the Methods section under Datasets and processing:

“In both cases, ChromaFactor was applied to analyze chromatin conformation at individual genomic loci (10Mb or smaller) rather than genome-wide to focus investigation of local chromatin organization and transcriptional activity.”

4. Besides visualization through UMAPs colored by the predominant component (Figures 2a and 3e), does matrix H capture known biological variations among cells?

Thank you for your question. Yes, the H matrix captures biological variation beyond what is shown in the UMAP visualizations. This query prompted us to expand our figures to include investigation of the H matrix itself. We now demonstrate the unique information in the H matrix in two ways:

1. We have added a supplementary figure (**Supplementary Figure 5)** showing a heatmap of the H matrix with cells labeled by transcriptional state. This visualization shows that cells with similar component weights cluster together, and there is evidence that the weights of some components correlate with transcriptional activity.
2. More quantitatively, our random forest analysis (**Figure 3b**) successfully predicts transcriptional state using only the H matrix as input, achieving an accuracy of 67.4% on a balanced sample of cells for some genes. This performance provides strong evidence that the weight matrix captures biologically meaningful variation in chromatin organization between transcribing and non-transcribing cells.

5. Although the matrix decomposition step only requires contact matrices, the method's primary application seems to rely on transcriptional profiles from corresponding cells. Some clarification and discussion of the limitation is needed.

Thank you for raising this important point about ChromaFactor's relationship to transcriptional data. While we demonstrate ChromaFactor's utility using datasets with matched transcriptional data, the method itself is unsupervised and operates solely on contact matrices. The transcriptional data in our examples serves as validation that the identified components are biologically meaningful. The absence of matched transcriptional data does not limit ChromaFactor's ability to identify biologically meaningful structural patterns; it only limits our ability to directly interpret these patterns in the context of single-cell transcriptional variation.

ChromaFactor can be valuable even without matched transcriptional data by identifying associated regulatory elements and mechanisms in an unbiased way. For instance, an analysis we did in response to Comment #7 (below) demonstrates how ChromaFactor’s outputs can also be interpreted using bulk epigenetic data, such as ChIP-seq for architectural proteins. More broadly, templates may highlight boundaries, compartments, or other structural features that could nominate regions for further investigation. This is particularly valuable since the vast majority of available single-cell chromatin conformation data lacks matched transcriptional data. In such cases, ChromaFactor can generate testable hypotheses about regulatory mechanisms by identifying subpopulations of cells with distinct chromatin organization patterns.

6. I cannot find multiple hypothesis corrections of all the reported p-values.

Thank you for raising this important point about multiple hypothesis correction. We have implemented Benjamini-Hochberg false discovery rate (FDR) correction for all statistical tests in our analysis. For the Abd-A locus analysis (Figure 3d), where we perform 20 tests comparing component weights between transcribing and non-transcribing cells, we applied FDR correction. After controlling for multiple testing with FDR < 0.1, eighteen components showed significant differences, with most showing strong significance (FDR < 0.001). For the HLCS locus analysis (Figure 4b), using the same FDR correction approach, five components remained significant (FDR < 0.1).

We have updated the Methods section to explicitly describe our multiple testing correction approach:

“To account for multiple comparisons when testing k components, we applied Benjamini-Hochberg false discovery rate (FDR) correction. Components with FDR < 0.1 were considered significant. For the Abd-A locus analysis, eighteen components showed significant differences after FDR correction (FDR < 0.05 for most components). For the HLCS locus analysis, five components showed significant differences after FDR correction (FDR = 0.021-0.091).”

We have updated all relevant figure legends and text to reflect these FDR-corrected p-values.

7. On the second paragraph of Page 3, it is stated that “we find that several templates resemble chromatin boundaries”. It is unclear to me what boundaries they are or how this claim is supported.

We thank the reviewer for this important point about clarifying chromatin boundaries. We have now added quantitative validation of these boundaries through analysis of insulation scores across templates and examination of architectural protein binding. Specifically, we show that template boundaries (regions of high insulation) consistently coincide with binding sites for key architectural and insulator proteins CTCF, Rad21, and CP190. This analysis is now presented in **Supplementary Figure 4**, which demonstrates the correspondence between template boundaries and protein binding sites. Most boundaries show concurrent binding of all three proteins, supporting their identification as genuine chromatin boundaries. This additional analysis provides strong support for our interpretation of these features as chromatin boundaries and offers insight into how different cell subpopulations might establish alternative boundary configurations. We updated the main text as follows:

"To quantitatively validate these boundaries, we computed insulation scores across each template and examined their correspondence with known architectural proteins (Supp. Fig. 4). Analysis of ChIP-seq data for boundary-associated proteins (CTCF, cohesin subunit Rad21, and insulator CP190) revealed strong colocalization with template boundaries in templates 0, 1 and 5. Most boundaries show concurrent binding of all three proteins, though some locations exhibit CTCF and CP190 binding without Rad21. This pattern suggests that different cell subpopulations may rely on different combinations of these architectural proteins to establish alternative boundary configurations, potentially explaining the distinct boundary patterns observed across templates."

We additionally added arrows in Figure 2 to clarify boundary locations.

8. Regarding transcription prediction using random forest models, it is unclear what the dotted line in Figure 3b is and what a random baseline accuracy is. Also, the current performance might be limited by the default parameters. Would the performance improve with parameter tuning with nested cross-validation? Would a regression model result in better predictive power?

Thank you for these questions about the random forest analysis. We have updated Figure 3b to clarify that the dotted line represents random chance performance (accuracy = 0.5). This 0.5 baseline represents true random performance because we deliberately constructed balanced datasets with equal numbers of transcribing and non-transcribing cells for each gene, as described in the Methods. Without this balancing, random performance would be biased toward the majority class, potentially obscuring the biological signal. A note on this point was clarified in the main text.

Regarding parameter tuning and model choice: We note that the random forest is used primarily as a tool to identify which templates are biologically relevant to transcription, rather than as an optimized classifier. The transcription data in our datasets is binary (on/off), making classification rather than regression the appropriate choice. We have expanded the text in the Results section to better explain this point.

Our key finding - that specific templates show predictive power for Abd-A while performing near random for unrelated genes - is robust to parameter choices, as demonstrated by two key points. First, this specificity is confirmed through permutation testing, where randomly shuffled transcription labels reduce performance to random chance (0.51 ± 0.01, p < 2.2×10^-16) for Abd-A while control genes like inv_Intron remain at baseline performance (0.500 ± 0.01) (new **Supplementary Figure 6**). Second, the templates identified as important by the random forest overlap completely with those identified through independent t-tests (components 0, 1, 5, 14, and 19, p < 0.001), suggesting that our findings reflect genuine biological signals rather than methodological artifacts.

9. Associating transcription with contact patterns is the most interesting part, though such analysis's advantages or biological insights are not adequately explained or validated. Are these identified components representing general cell state changes or specific to the expression of Abd-A and HLCS? What does “a steep shift in the directionality index” inform regarding transcriptional regulation (Figure 4c)?

Thank you for your comments about the connection between transcription and contact patterns. We have substantially expanded our analysis to address these points:

1. Regarding the advantages and biological validation of the transcription analysis:

We demonstrate the method's advantages and biological significance through two complementary analyses in different systems. In the human system, we expanded our analysis to examine multiple genes within a 10Mb region (new **Supplementary Figure 8**), revealing how template analysis can distinguish different regulatory strategies that appear similar in bulk analysis. For example, while BACE2 and SON show comparable changes in their bulk delta contact maps, template analysis reveals associations with distinct templates - the activity at BACE2 requires a minimal contribution from one component, while SON's activity depends on a subset of cells with high weights in a different component.

Additionally, in the Drosophila system (Abd-A locus), we validate the biological significance of our templates by demonstrating that template boundaries strongly colocalize with architectural proteins (CTCF, Rad21, and CP190) (new **Supplementary Figure 4**). This analysis reveals that different cell subpopulations may rely on different active sets of architectural proteins binding in different locations to establish alternative boundary configurations, providing mechanistic insight into how chromatin organization relates to transcriptional regulation.

2. Regarding whether components represent general cell states or gene-specific changes:

Our human locus analysis addresses this question. We now analyze five genes (SON, RUNX1, SIM2, ETS2, and BACE2) within the same 10Mb region. We find that different genes use distinct sets of significant components despite their proximity. This suggests that components can capture gene-specific regulatory mechanisms rather than just general cell state changes.

3. Regarding the interpretation of directionality index shifts: we have expanded our discussion and literature support of directionality index, noting that directionality index change denotes domain boundaries, and the loss or gain of insulation across a locus may influence the interaction frequency between enhancers and their target genes, impacting transcription.

**Reviewer #2:**

In this manuscript, the authors propose an NMF-based framework, ChromaFactor, to decompose single-cell datasets into different components and identify important cells influencing cellular phenotypes. By applying ChromaFactor to two single-cell genome folding datasets, it reveals patterns and structures, linking these to functional outputs like nascent transcription. The proposed framework is easy to understand, and the GitHub link is provided. However, I have the following concerns about the manuscript.

We thank the reviewer for their positive feedback about how we communicated this research project, and we hope that our revision fully addresses the concerns raised.

1. There is inconsistency in the notation used for cells between different sections of the manuscript. For example, 'n' is used for cells in the first paragraph of the Results section, while 'm' is used in Figure 1. Additionally, the number of cells in the Mateo dataset is unclear. it is 19,103 or 16,320? This discrepancy should be clarified to avoid confusion.

Thank you for requesting this important clarification. We have made the following changes:

1. To improve notation consistency, we now use 'b' for genomic bins (previously 'n'), 'n' for number of cells, and maintain 'k' for components.
2. The initial dataset contained 19,103 cells, from which we excluded 2,783 cells (those with >80% missing coordinates), resulting in 16,320 cells used in the final analysis. We have updated all relevant sections of the manuscript to consistently report this final number and expanded an explanation in the Methods section.

2. The criteria for determining the number of components (k) are not well explained. From Supplementary Figure 1, it is difficult to discern why k=20 was chosen. It would be better to show the performance of the framework with different numbers of components to provide a clearer justification for selecting k=20.

We thank the reviewer for this important point about k selection. To address the problem, we have substantially expanded our approach to determining the optimal number of components through a new comprehensive evaluation framework as a part of ChromaFactor: *KSelector*. Rather than relying solely on reconstruction error, we now consider multiple complementary metrics:

1. Reconstruction error
2. Component stability across multiple random initializations
3. Component redundancy to evaluate if additional components capture unique patterns
4. Computational efficiency (runtime)
5. Biological significance through transcription prediction (when matched labels are available)

Our new framework allows users to balance multiple criteria when selecting k for their specific application. This class evaluates multiple ChromaFactor instances across a range of k choices and random initializations. The results of all metrics are logged to enable reproducible analysis. The framework additionally plots all results and suggests an optimal k by considering which selection produces the best average result across these five metrics. Users may also select k based on the priorities of their specific project using the provided output plots.

We have added a new section of the Methods describing this framework and a new supplementary figure showing how these metrics vary across different k values for the *Mateo et al.* dataset (**Supplementary Figure 2**). We deeply thank the reviewer for this suggestion as this addition has strengthened the manuscript considerably.

3. The classification accuracy shown in Figure 3b is close to random, making it challenging to identify which feature is more important. Additional baseline methods are needed to evaluate the framework's performance.

We appreciate this observation and would like to clarify the interpretation of the random forest analysis. While the random forest does serve as a predictive model, its primary purpose in our analysis is not to optimize prediction accuracy, but rather to serve as one of two complementary approaches to identify which templates are biologically relevant to transcription. The pattern in Figure 3b demonstrates the specificity of our templates. They show predictive power (0.674) specifically for Abd-A transcription while performing at random for unrelated genes, which is what we would expect if capturing conformations specific to Abd-A regulation.

To further validate this finding, we conducted a targeted permutation analysis on both our strongest signal (Abd-A transcription) and a negative control (inv_Intron transcription). For each gene, we performed 10 independent permutations where transcription labels were randomly shuffled while maintaining the same proportion of transcribing to non-transcribing cells. For each permutation, we trained a new random forest model using identical parameters to our original analysis on the shuffled labels. When transcription labels are randomly permuted, the predictive performance for Abd-A drops from 0.674 to random chance (mean accuracy 0.498 ± 0.003, p < 2.2×10^-16). For inv_Intron, the permuted results showed performance at theoretical random chance (0.500 ± 0.002), slightly below its original performance (0.511) but both effectively at random performance levels. This permutation analysis demonstrates that the predictive performance for Abd-A transcription is not arising by chance, while confirming that performance on unrelated genes like inv_Intron represents baseline random prediction, validating the biological specificity of our templates. We have added **Supplementary Figure 6** showing permutation testing results.

Finally, the templates identified as most important by the random forest analysis (components 0, 1, 5, 14, and 19) are the same templates that show significant differences between transcribing and non-transcribing cells in our t-test analysis (p < 0.001 for all five components). Supporting evidence from two different statistical approaches, one supervised (random forest) and one unsupervised (t-tests), strengthens our confidence that the method identifies biologically relevant templates.

4. This framework is not a standard NMF. It is important to provide more information in the Methods section to explain how different template matrices are learned with the same weight matrix. Specifically, the process of "unraveling the 3D input matrices into 2D vectors suitable for NMF" needs a detailed explanation to enhance reproducibility and understanding.

Thank you for this important point. We have substantially expanded the "Non-negative matrix factorization (NMF) and ChromaFactor application" section of the Methods to provide a detailed technical explanation of how ChromaFactor adapts standard NMF for single-cell 3D chromatin conformation data. The revised section now includes details of how spatial relationships are preserved during matrix transformation and mathematical formalization of the tensor reshaping process.

5. From a computational perspective, the novelty of the proposed framework appears limited.

ChromaFactor deliberately relies on mathematical simplicity for biological insight. We view this simplicity as a key strength of our method for three main reasons. First, ChromaFactor's computational accessibility, requiring only standard libraries and minimal setup, makes it immediately usable. This contrasts with methods requiring specialized computational infrastructure, GPU support, or complex installation procedures, enabling rapid application to new datasets on a laptop. Second, the method's interpretability stems directly from its mathematical simplicity. The NMF components have clear biological interpretations, unlike some more complex black-box approaches, and the relationship between templates and cell behaviors directly connects how specific subpopulations contribute to observed phenomena. This transparency is useful for rapidly generating new testable biological hypotheses. Third, while computationally straightforward, ChromaFactor is the first method to explicitly connect transcriptional state to 3D conformations across single-cell populations. This is particularly important because the key challenge with this type of data is that an extreme minority of cells demonstrate differences in relation to transcriptional differences. There are multiple sources of complexity that make this a poor candidate for more complex approaches like deep learning: chromatin's dynamic nature means relevant conformations may be transiently present and missed in most cells (noise in input), nascent RNA detection is not guaranteed in all cells (noise in output), and the single-molecule nature of these experiments makes measurement errors and data dropout extremely common (noise in experiment). The simplicity of our method allows us to bridge single-cell heterogeneity with functional outcomes despite these challenges, which is enabled by, rather than limited by, the method's simplicity. Finally, as noted in our responses to Reviewer 1, ChromaFactor is designed to be run on a single locus and does not require genome-wide data for estimating parameters or performing cross-validation.

**Reviewer #3:**

In the article "ChromaFactor: deconvolution of single-molecule chromatin organization with non-negative matrix factorization," Laura M. Gunsalus, Michael J. Keiser, and Katherine S. Pollard consider the topic of deconvolution of single-molecule chromatin organization with non-negative matrix factorization.

The authors address the problem that analysis of single-molecule data is hampered by extreme yet inherent heterogeneity, making it challenging to determine the contributions of individual chromatin fibers to bulk trends. They mention that several computational methods have been developed in response to emerging single-cell imaging and high-throughput sequencing techniques to measure chromatin conformation. They claim that these works did not yet connect the behavior of individual cells to populations of similar conformations that are transcriptionally on or off.

The authors provide a computational approach based on non-negative matrix factorization that deconvolves single-molecule chromatin organization datasets into components and identifies subpopulations that correlate with cellular phenotypes.

We are glad that the method was presented clearly enough to enable the reviewer to provide this excellent summary of ChromaFactor.

It is not clear whether ChromoFactor enhances the large body of existing literature on single-cell chromatin architecture. A more thorough summary of the literature needs to be provided and compared and contrasted with ChromoFactor.

We appreciate the opportunity to clarify how our method builds on existing literature.

The traditional problem formulations in single-cell chromatin organization have focused on several key computational tasks:

1. Processing sparse data through denoising and imputation (e.g., HiCDiff, Higashi, SnapFISH-IMPUTE)
2. Identifying chromatin structures like loops, TADs, and compartments (e.g., SnapHiC, scGHOST)
3. Grouping similar cells through clustering and embedding (e.g., Higashi, Topic modeling)

ChromaFactor introduces a fundamentally different problem: identifying which specific cells drive the patterns we observe in bulk experiments. Many mechanistic claims in regulatory genomics are based on bulk patterns, yet we don't know if these structures exist in individual cells or are solely averaging artifacts. Moreover, when patterns do exist in single cells, determining which subset of cells exhibits them is crucial for understanding mechanisms; patterns driven by 10% versus 90% of cells suggest distinct regulatory models.

While existing computational methods perform well at their intended applications, they do not address this core question of linking individual cell behavior to population-level phenomena. For example, Rajpurkar et al. developed a deep learning approach to predict transcription from chromatin folding, and Zhan et al. developed methods to cluster conformations, but neither method identifies which specific cells drive the patterns we observe in bulk experiments.

We believe this new problem formulation is particularly relevant as the field progresses from technical data processing challenges toward understanding mechanistic relationships between chromatin structure and function. ChromaFactor's use of interpretable matrix factorization, rather than black-box deep learning approaches, makes it particularly well-suited for connecting population-level patterns to specific cellular behaviors.

Finally, ChromaFactor represents an initial step toward viewing single-cell chromatin measurements not as independent instances, but as snapshots of continuous, dynamic states. By identifying groups of cells that contribute to specific patterns, our approach begins to bridge the gap between static single-cell measurements and the dynamic nature of chromatin organization. This perspective will become increasingly important as the field moves toward understanding how chromatin structure evolves over time to influence cellular function.

In response to your suggestion, we expanded the literature review in the introduction to better contextualize ChromaFactor's unique contribution and expanded the discussion to explore how this new approach complements existing methods in the field.

It isn't easy to assess the significance of the method in the context of 3D genome biology research since only one biological example involving a single locus was provided and the utility of the reported result is not clear.

We appreciate the reviewer's concern about significance and utility. To address this, we have expanded our study to demonstrate ChromaFactor's application across multiple biological contexts and scales. We also emphasize now that the ability to be run on a single genomic locus (<10Mb) is a strength of ChromaFactor that enables it to be applied to imaging data from emerging technologies that only capture information for a single locus.

ChromaFactor addresses a fundamental challenge in single-cell chromatin analysis: while bulk experiments may show clear patterns of chromatin organization, these patterns are nearly impossible to discern in individual cells. Our method provides three key utilities:

1. Identification of Functional Subpopulations: ChromaFactor may reveal which specific cells drive the patterns seen in bulk data. For example, we show that fewer than 15% cells are responsible for the strong boundary patterns associated with Abd-A transcription. We find a similar result at the HLCS locus.
2. Discovery of Different Regulatory Strategies: As demonstrated in our new analysis (**Supplementary Figure 8**), our method can distinguish how different genes achieve similar bulk contact differences through distinct mechanisms. We investigate a 10Mb region containing multiple genes (SON, RUNX1, SIM2, ETS2, and BACE2), and find that similar bulk contact changes can arise through distinct template combinations. While BACE2 and SON exhibit similar population-level contact changes in transcribing and non-transcribing cells, our analysis shows that BACE2 transcription requires a minimal threshold contribution from one component, while SON's activity is driven by a small subset of cells with exceptionally high weights in a different component.

3. Mechanistic Insights: By connecting template patterns to architectural proteins and transcriptional state, ChromaFactor helps explain how chromatin organization relates to function. Our new analysis shows how different combinations of CTCF, Rad21, and CP190 binding correspond to template boundaries (**Supplementary Figure 4**), providing mechanistic insight into how chromatin organization is established and maintained in different cell subpopulations.

These analyses demonstrate ChromaFactor's broad utility for deconvolving chromatin organization across:

- Different model systems (Drosophila and human)
- Multiple genomic scales (from 100kb to 10Mb regions)
- Diverse biological phenomena (architectural protein binding, gene-specific regulation)
- Distinct regulatory mechanisms (threshold-dependent vs population-driven transcription)

If the reviewer feels additional analysis would be valuable, we could also analyze another dataset.
